# Role of Stromal Paracrine Signals in Proliferative Diseases of the Aging Human Prostate

**DOI:** 10.3390/jcm7040068

**Published:** 2018-04-02

**Authors:** Kenichiro Ishii, Sanai Takahashi, Yoshiki Sugimura, Masatoshi Watanabe

**Affiliations:** 1 Department of Oncologic Pathology, Mie University Graduate School of Medicine, Tsu, Mie 514-8507, Japan; mawata@doc.medic.mie-u.ac.jp; 2 Department of Nephro-Urologic Surgery and Andrology, Mie University Graduate School of Medicine, Tsu, Mie 514-8507, Japan; sugimura@clin.medic.mie-u.ac.jp; 3 Laboratory for Medical Engineering, Division of Materials Science and Chemical Engineering, Graduate School of Engineering, Yokohama National University, Yokohama, Kanagawa 240-8501, Japan; sanai.takahashi@gmail.com

**Keywords:** proliferative diseases of the aging human prostate, epithelial–stromal interactions, stromal remodeling, stromal paracrine signals, carcinoma-associated fibroblast-derived exosomal microRNAs

## Abstract

Androgens are essential for the development, differentiation, growth, and function of the prostate through epithelial–stromal interactions. However, androgen concentrations in the hypertrophic human prostate decrease significantly with age, suggesting an inverse correlation between androgen levels and proliferative diseases of the aging prostate. In elderly males, age- and/or androgen-related stromal remodeling is spontaneously induced, i.e., increased fibroblast and myofibroblast numbers, but decreased smooth muscle cell numbers in the prostatic stroma. These fibroblasts produce not only growth factors, cytokines, and extracellular matrix proteins, but also microRNAs as stromal paracrine signals that stimulate prostate epithelial cell proliferation. Surgical or chemical castration is the standard systemic therapy for patients with advanced prostate cancer. Androgen deprivation therapy induces temporary remission, but the majority of patients eventually progress to castration-resistant prostate cancer, which is associated with a high mortality rate. Androgen deprivation therapy-induced stromal remodeling may be involved in the development and progression of castration-resistant prostate cancer. In the tumor microenvironment, activated fibroblasts stimulating prostate cancer cell proliferation are called carcinoma-associated fibroblasts. In this review, we summarize the role of stromal paracrine signals in proliferative diseases of the aging human prostate and discuss the potential clinical applications of carcinoma-associated fibroblast-derived exosomal microRNAs as promising biomarkers.

## 1. Introduction

The prostate is a male accessory sex gland found only in mammals. Its main function is to produce a major proportion of the seminal fluid. Androgens are essential for the development, differentiation, growth, and function of the prostate [1]. 

**Figure 1:**
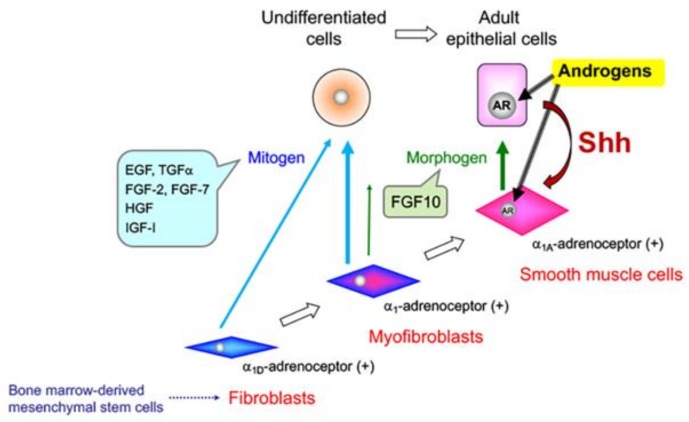
Epithelial–stromal interactions in the developing prostate. Bone marrow-derived mesenchymal stem cells may differentiate into fibroblasts, myofibroblasts, and smooth muscle cells in prostatic stroma. Fibroblasts and myofibroblasts secrete mitogens to stimulate the proliferation of undifferentiated cells. Smooth muscle cells secrete morphogens to maintain the functional differentiation of adult epithelial cells. AR: androgen receptor; Shh: sonic hedgehog.

The prostate develops from the urogenital sinus and is induced by embryonic mesenchyme, fibroblasts, and myofibroblasts during its development and differentiation [2]. Ductal morphogenesis, epithelial differentiation, and proliferation/apoptosis are regulated by androgens acting through the stromal androgen receptor (AR). As summarized in Figure 1, androgen-mediated stromal paracrine signals support normal epithelial differentiation and function [3]. As stromal paracrine signals for epithelial cells, stromal components such as fibroblasts, myofibroblasts, and smooth muscle cells secrete a number of growth factors, cytokines, extracellular matrix (ECM) proteins, and microRNAs (miRNAs). Thus, androgen-mediated epithelial–stromal interactions play crucial roles in ductal morphogenesis and epithelial differentiation in the development of the prostate [4,5].

In the adult prostate, stroma composed of smooth muscle cells interact with epithelial cells. These epithelial–stromal interactions maintain the functional differentiation and growth/quiescence of epithelial cells [6]. In adults, androgens act on AR-positive epithelial cells to establish their functional differentiation into luminal secretory epithelial cells [7]. Moreover, epithelial AR is responsible for maintaining the differentiated phenotype and overall homeostasis of the prostate glands [6].

Androgens also act on epithelial cells to produce growth factors, cytokines, and miRNAs that mediate stromal functions, including proliferation and differentiation [8,9,10]. On the other hand, androgens act on smooth muscle cells to produce a number of morphogens critical for maintaining the differentiation and supporting the proliferation of epithelial cells [11]. Furthermore, under the influence of androgens, stromal cells produce mitogens that stimulate the proliferation of epithelial cells [12]. Thus, these androgen-mediated paracrine effects between epithelial and stromal cells maintain homeostasis in the adult prostate [13,14]. An interesting feature of the adult prostate is that the tissue does not actively proliferate, and there is little cellular turnover even in the presence of growth-stimulating androgens. Moreover, the incidences of proliferative diseases of the aging human prostate, including benign prostatic hyperplasia (BPH) and prostate cancer (PCa), apparently increase with advancing age, despite decreasing androgen levels [15].

The androgen status affects prostatic structure and components. In the absence of androgens, prostatic structural regression is mainly attributed to a functional decrease in the secretory activity of luminal epithelial cells and to a reduction in the number of luminal epithelial cells by apoptosis [16,17]. Structural alterations also occur in the stroma following castration and are characterized by the replacement of smooth muscle cells by fibroblasts or myofibroblasts, called stromal remodeling [18,19]. Evidence suggests that castration-induced stromal remodeling is accompanied by functional transformation of the prostatic stromal environment [20]. In luminal epithelial cells, prostatic secretory functions and numbers are dramatically decreased in the absence of androgen, while basal epithelial cells remain intact to form a continuous layer lining the atrophied prostate acini in the absence of androgen [18,19]. Interestingly, this regression process after castration is reversible, because androgen replacement leads to complete regeneration of the prostate with secretory function [21,22,23,24]. The mechanisms of prostatic regeneration may include the participation of prostate stem/progenitor cells with self-renewing ability [25,26]. For example, a single transplanted stem cell is sufficient to regenerate the prostate in vivo [24]. 

## 2. Benign Prostatic Hyperplasia (BPH)

BPH is a common prostatic disease in elderly males. The incidence of BPH increases with age and more rapidly than that of PCa [27]. Notably, most PCas arise in the prostate concomitant with BPH elsewhere [28]. 

BPH is frequently observed in middle-aged and elderly males; its prevalence is only 8% during the fourth decade of life, but more than 70% during the seventh decade [29]. Its major manifestations are lower urinary tract symptoms. Because it is benign, severe complications are rare. However, from the standpoint of preserving quality of life, it is essential to alleviate the symptoms of BPH. Regarding the natural history of BPH, Berry et al. reported that prostate weight increases sharply as androgen levels increase before adolescence, although the rate of increase becomes much slower after the age of 30 years [29]. However, prostate weight begins to increase again over the age of 60 years, with a sharp increase in the 70s. In an analysis of Japanese cases reported by Fujikawa et al., prostate weight tended to increase sharply from birth to the third decade of life and thereafter increased gradually, with no sharp increase after age 60 [30]. These two reports suggest that prostate weight is lower in Japanese than in Western individuals, and that prostate weight gain is less in Japanese males. 

With regard to the size of the prostate, Ishigooka et al. reported a correlation between prostate volume and increased fibrous tissue in a histological examination of surgical specimens obtained by transurethral resection of the prostate (TURP), suggesting that fibrous tissue plays an important role in BPH [31]. Ichiyanagi et al. performed a histological examination of TURP specimens and found that the proportions of stroma and gland cavities in the prostate differed little between patients with and those without disturbance of the lower urinary tract passage, while the proportion of smooth muscle in prostatic stroma was significantly lower in patients with a disturbance in the lower urinary tract passage [32]. 

## 3. Alteration of Stromal Structure by α_1_-Adrenoceptor Antagonists (α_1_-Blockers)

At present, BPH is treated by drug therapies, surgery (primarily TURP), or less-invasive therapies using lasers, stents, and other means. α_1_-adrenoceptor antagonists (α_1_-blockers) are often used as first-line drugs [33]. Even at present, 20 years since their clinical use was introduced, α_1_-blockers are considered useful in the treatment of BPH. They suppress functional contraction of prostatic smooth muscle cells by blocking signaling from the α_1_-adrenoceptors distributed in this muscle. α_1_-blockers are highly efficacious in alleviating lower urinary tract symptoms and are therefore frequently used clinically. 

Smith et al. found that the contractility of prostatic smooth muscle cells decreased following the inhibition of adrenergic receptor signaling by α_1_-blockers [34]. Their finding suggests that α_1_-blockers induce dedifferentiation of smooth muscle cells into fibroblasts and myofibroblasts and may thus induce alteration of tissue architecture. Moreover, Justulin et al. reported that when rats were given repeated doses of doxazosin, an α_1_-blocker, they exhibited an accumulation of collagen fibers in the prostatic stroma and a reduction in smooth muscle cells [35]. Based on this finding, they suggested the possibility that continued oral treatment with doxazosin would alter stromal structure and components, reducing the efficacy of α_1_-blockers [35]. These views were endorsed by the findings of our study, in which treatment with α_1_-blockers resulted in alteration of stromal structure and components [36]. However, the mechanisms responsible for such changes have not yet been elucidated. Justulin et al. suggested that transforming growth factor (TGF)-β is an intracellular transmitter potentially involved in these stromal alterations [35]. TGF-β is a cytokine with diverse functions. In the ECM, fibroblasts, osteoblasts, odontoblasts, and other types of cells induce the expression of *COL1A1* and *COL1A2* (genes encoding type I collagen) in the presence of TGF-β, resulting in the stimulation of type I collagen formation [37]. 

## 4. Aberrant Activation of Epithelial-Stromal Interactions in BPH

**Figure 2:**
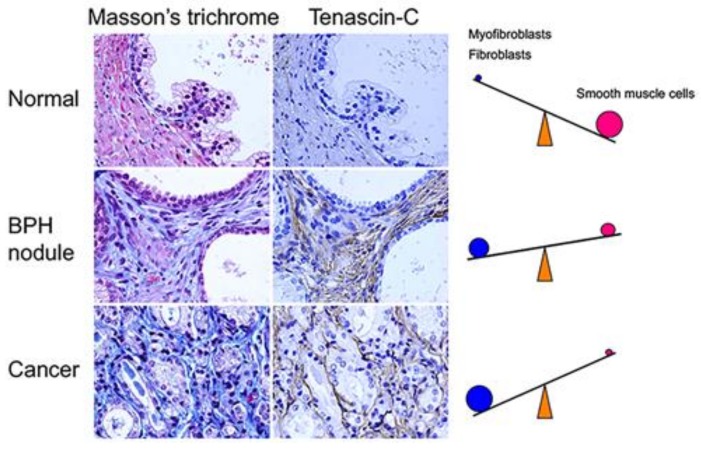
Stromal changes associated with proliferative diseases of the aging human prostate. Surgical tissue specimens obtained from patients with BPH or PCa were histologically stained with Masson’s trichrome and immunostained with an anti-tenascin-C antibody. Masson’s trichrome stained smooth muscle cells pink and fibroblasts/myofibroblasts blue. BPH: benign prostatic hyperplasia; PCa: prostate cancer. Magnification 400×.

Deregulation of epithelial–stromal interactions is considered to be responsible for the initiation and/or promotion of proliferative diseases of the aging human prostate, including BPH and PCa [38,39]. As shown in Figure 2, stromal nodules associated with BPH are composed of fibroblasts, myofibroblasts, and smooth muscle cells [40]. As a potential developmental mechanism of BPH, Pradhan et al. reported that nodular hyperplasia originates as an early stromal nodule, usually by the side of the urethra, stimulating the duct in its vicinity to proliferate [41]. 

According to the hypothesis proposed by McNeal, stromal nodules are the result of the reappearance of embryonic ductal morphogenesis, and BPH develops through a change in stromal differentiation into the fetal phenotype [42]. Norman et al. suggested that in mice, the fetal stroma reacts with post-developmental prostate epithelia, resulting in the formation of new prostate tissue [43]. Their report favors the hypothesis of McNeal and suggests that various growth factors, cytokines, ECM proteins, and miRNAs involved in aberrant activation between epithelial and stromal cells are related to the onset of BPH. 

## 5. Effects of Sex Steroid Hormone Status on Basal Epithelial Cell Behavior in the Prostate 

The prostate contains two major epithelial cell types: luminal and basal epithelial cells. Basal epithelial cells are critical for maintaining ductal integrity and regulating both the survival and apoptosis of luminal epithelial cells [44,45,46,47,48]. Moreover, prostate progenitor and stem cells have been identified within the basal compartment [49]. 

An increase in basal epithelial cells is referred to as basal cell hyperplasia, which is defined as basal cell proliferation composed of two or more layers of small cells with scant cytoplasm presenting as glands or solid nests [50]. Basal cell hyperplasia is occasionally a component of untreated BPH, which arises in the transition zone of the human prostate [50,51,52]. Alteration of the proliferation of basal epithelial cells in the peripheral zone was suggested to be involved in the initiation and early progression of PCa [53]. Clinically, androgen ablation by antiandrogen therapy for patients with advanced PCa results in basal cell hyperplasia with variable focal squamous metaplasia localized diffusely throughout benign prostate tissue [54].

Several studies have reported that androgen ablation leads to the death of luminal secretory epithelial cells, while basal cells, an AR-negative population, survive [18,19]. The androgen concentration in the hypertrophic human prostate decreases significantly with age [15]. In the dorsolateral prostate (DLP) of senescence-accelerated mice deficient in androgen, stromal fibrosis, the presence of atypical glandular epithelial cells, and cribriform glandular deformities were observed as age-related alterations [55]. In the canine prostate, the effects of androgen ablation on basal epithelial cells and luminal epithelial cells are associated with a marked increase in the stromal fibromuscular compartment, which demonstrates impaired differentiation [18]. The age-related expansion of proliferating acinar basal epithelial cell populations, mediated by sex steroid hormones, is a key factor in the pathogenesis of canine prostatic hyperplasia [56]. These findings indicate that an aberrant proliferation of epithelial cells may be related to the concentration of androgen in the prostate. 

In our lab, Kato et al. demonstrated that the number of basal epithelial cells in the mouse prostate was affected by changes in androgen status [19]. Interestingly, the number of basal epithelial cells was increased in the absence of androgen and returned to baseline levels following androgen replacement. We propose that these proliferative alterations observed in the regressed prostate may compensate for the loss of luminal epithelial cells to maintain residual ductal components. Androgen replacement after castration drives complete regeneration of the gland, which is derived entirely from the proliferation and differentiation of surviving cells, including basal epithelial cells [57].

In the castrated mouse prostate, the levels of specific growth factors are increased in the setting of androgen withdrawal [19,58,59,60]. To examine how specific growth factors affect prostatic structure, we analyzed androgen-status-dependent changes in the gene expression of growth factors in involuted mouse prostates [19]. After castration, the mRNA expression levels of specific growth factors, such as *Fgf2*, *Fgf7*, *Hgf*, *Tgfa*, and *Tgfb*, were relatively abundant in whole mouse DLPs. In embryos, these growth factors stimulate the proliferation of epithelial cells and play an important role in prostate development and ductal morphogenesis [61,62]. In organ culture experiments, abnormal basal epithelial proliferation was recapitulated in the absence of dihydrotestosterone (DHT). The proliferation of basal epithelial cells in the absence of DHT was suppressed by treatment with a fibroblast growth factor (FGF) receptor inhibitor (PD173074). Moreover, FGF2 treatment directly stimulated the proliferation of basal epithelial cells. Finally, we concluded that the FGF2–FGF receptor signaling cascade in the prostate gland may be one pathway that stimulates the proliferation of basal epithelial cells in the absence of androgens [19].

In the prostate, squamous metaplasia, which involves an increase in the number of basal epithelial cells [63], is induced by estrogens, including 17β-estradiol (E_2_), diethylstilbestrol (a synthetic estrogen), and bisphenol A (an endocrine-disrupting chemical), which has weak estrogenic activity [64,65,66,67]. Estrogen action in the prostate is mediated through estrogen receptors α (ERα) and β (ERβ). In particular, estrogen-induced squamous metaplasia requires ERα signaling in both epithelial and stromal cells [63]. In neuroblastoma cells, E_2_ induced synthesis of transforming growth factor α (TGFα) via ERα, and TGFα in turn, induced cell proliferation [68]. In another report, long-term treatment with gonadal steroids, testosterone, and E_2_ induced dysplasia and enhanced TGFα and epidermal growth factor (EGF) receptor expression in the normal rat prostate [69]. Regarding the role of TGFα, we demonstrated that the number of basal epithelial cells in the prostate of TGFα-overexpressing mice was increased [70]. These reports support that TGFα may be a second messenger in estrogen signaling, mediating estrogen-induced basal epithelial cell growth in the prostate. 

## 6. Prostate Cancer (PCa)

The number of males diagnosed with PCa is increasing all over the world [71]. Most patients with early-stage PCa can be treated by the appropriate therapy such as radical prostatectomy or irradiation. On the other hand, androgen deprivation therapy (ADT) is the standard systemic therapy given to patients with advanced PCa. ADT induces temporary remission, but the majority of patients eventually progress to castration-resistant PCa (CRPC), which is associated with a high mortality rate [72,73]. CRPC, a heterogeneous disease, exhibits varying degrees of androgen sensitivity. Once PCa cells lose their sensitivity to ADT, effective therapies are limited. The progression of PCa cells from local invasion to distant metastasis and androgen insensitivity may be influenced by alterations in the tumor microenvironment, AR mutations, overexpression of growth factors and their receptors, and secretion of ECM proteins and miRNAs, leading to selection of cells with higher aggressive potential [10,74]. Understanding the biological changes that occur in the androgen-insensitive state may help identify new pathways as drug targets. 

Acute loss of AR function after ADT is associated with not only apoptosis and a reduction in prostate-specific antigen (PSA) secretion, but also promotion of AR-independent growth in PCa cells. Disruption of androgen signaling by ADT may result in deregulation of cell cycle control, which could contribute to carcinogenesis [75]. Nelson et al. described four molecular-state frameworks for AR activation in PCa after ADT as follows: state 1, endocrine androgen dependent and AR dependent; state 2, intracrine androgen dependent and AR dependent; state 3, androgen independent and AR dependent; and state 4, androgen independent and AR independent [76]. State 4 is considered the fatal stage, at which AR signaling is abolished, and neuroendocrine (NE) differentiation occurs. Burchardt et al. reported that LNCaP cells inoculated into castrated mice induced a significant increase in NE cells compared with inoculation into intact mice [77]. Although the mechanism of NE transdifferentiation induced by androgen deprivation of AR-dependent PCa cells has been investigated, the mechanism mediating NE differentiation is still not clear [78].

## 7. Tumor–Stromal Interactions in PCa

In adult prostate homeostasis, epithelial–stromal interactions maintain functional differentiation and growth/quiescence [79]. In contrast, the deregulation of epithelial–stromal interactions is considered to play a critical role in the initiation and/or promotion of carcinogenesis [38,39]. In the tumor microenvironment, aberrant activation between cancer cells and stromal cells significantly contributes to the progression of human cancers including prostate, breast, and colon [80,81,82,83,84].

Solid tumors are highly complex and heterogeneous and are composed of epithelial cancer cells infiltrating into the surrounding tumor stroma, called reactive stroma and comprising carcinoma-associated fibroblasts (CAFs) [85,86]. PCa is interesting because of the multi-focal and heterologous progression of primary tumors [87]. Recent in vivo studies have demonstrated that the heterogenous stromal compartment of the prostate contains multiple populations of fibroblasts that are associated with tumorigenesis [88,89]. Clinically, reactive stromal grading in radical prostatectomies or biopsies is a predictor of recurrence; a high reactive stromal grade is associated with lower biochemical recurrence-free survival rates compared with a low grade [90,91]. 

CAFs surrounding cancer cells secrete a number of growth factors, cytokines, ECM proteins, and miRNAs to support the survival and proliferation of cancer cells in a paracrine fashion [39,92]. Tsunoda et al. reported that the expression of periostin, a stromal biomarker in PCa, in the CAFs of PCa tissues was increased during the early stages of the disease (Gleason score 6–7) and was significantly correlated with the degree of malignancy [93]. Up-regulation of collagen I, tenascin-C, and TGF-β has been observed in PCa specimens [85]. In addition, the phenotype of cultured prostate CAFs expressing high levels of CD90, a marker of mesenchymal stem cells (MSCs), was associated with greater tumor promotion than that of cells with low CD90 expression [94]. CAFs with high CD90 levels expressed higher levels of many genes associated with tumor promotion, including TGF-β, the angiogenic factors vascular endothelial growth factor (VEGF) and FGF2, and the cytokines interleukin (IL)-6 and chemokine (C-X-C motif) ligand 12. Several studies have shown that CAFs isolated from PCa specimens are strongly heterogenous and have specific biochemical characteristics [95,96,97,98]. In our lab, we reported that primary cultured PCaSC-8 and/or PCaSC-9 cells derived from different human PCa specimens displayed a significantly higher mRNA expression of *COL1A1*, *TNC*, *EGF*, *FGF2*, *FGF7*, *HGF*, and *IGF1* [96]. The role of ECM proteins in tumorigenesis includes effects on epithelial polarity and angiogenesis [99]. In contrast, CAF-derived growth factors are predominantly stimulators of cancer cell proliferation and play a part in promoting the carcinogenic process [100]. Importantly, the expression profiles of CAF-related genes were heterogenous between PCaSC-8 and PCaSC-9 cells, suggesting that the biochemical characteristics of different human PCa specimen-derived CAFs are strongly heterogenous. Previous studies showed differences in biochemical characteristics between human PCa specimen-derived CAFs and adjacent normal fibroblasts [95,98].

The adult prostate contains an abundance of stromal components, mainly consisting of well-differentiated smooth muscle cells. In prostate tumors, however, fibroblasts surrounding cancer cells are associated with not only the initiation of cancer cells, but also tumor growth and progression to androgen independence [101,102,103]. Olumi et al. demonstrated stroma-induced malignant transformation, indicating that fibroblasts surrounding epithelial cells play an important role in PCa development [38]. Other groups reported that co-inoculation of PCa cells with fibroblasts increased tumorigenicity and potentially angiogenesis promotion [84,101,104]. Therefore, the inhibition of tumor–stromal interactions may exert a synergistic effect with the suppression of cancer cell growth on tumor control.

During cancer progression, stromal changes result in a decreased prevalence of smooth muscle cells (Figure 2). Tumor stroma surrounding cancer cells is enriched in fibroblasts and myofibroblasts [105]. Several studies have demonstrated the presence of myofibroblastic cells with the capacity to contribute ECM proteins and collagenous components to the reactive stroma surrounding tumors [85,106,107]. Myofibroblasts, which share characteristics with fibroblasts and smooth muscle cells, are activated fibroblasts typically found at sites of pathologic tissue remodeling, such as wound healing [108,109]. In cancers, myofibroblasts in reactive stroma show irregularities in length and thickness compared with fibroblasts. 

## 8. PCa Cell Lines with Different Levels of Androgen Sensitivity

Decrease or loss of androgen sensitivity in PCa is a clinical concern. Many studies on CRPC have used androgen-insensitive PCa cell lines, such as PC-3 and DU145 cells, which do not express AR. These cell lines originated from highly anaplastic tumors from different metastatic sites such as the bone and brain [110,111]. Both the PC-3 and DU145 cell lines are strongly different in terms of aggressiveness compared with the androgen-sensitive AR-positive LNCaP line, which originated from lymph node metastasis [112]. Comparisons between the androgen-sensitive LNCaP and androgen-insensitive PC-3 and DU145 cell lines may not be relevant to the acquisition of androgen insensitivity in clinical PCa, because many clinical androgen-insensitive PCa cases express AR. A more precise model of clinical cancer requires, at the very least, an androgen-insensitive AR-positive cancer cell line. To compare the biochemical characteristics of androgen-insensitive and androgen-sensitive PCa cells, we generated three sublines from androgen-sensitive LNCaP cells: E9 and F10 cell lines (low androgen sensitivity) [113,114] and androgen-insensitive AIDL cells [115]. The parental LNCaP cells and the derived E9, F10, and AIDL cells express similar levels of AR protein, but androgen-dependent PSA secretion is only detected in LNCaP cells [116]. Moreover, we have shown that recombination of E9 cells or AIDL cells with embryonic rat urogenital sinus mesenchyme (UGM) promoted tumor progression in vivo even under androgen ablation [116].

## 9. Origins of Cell Populations Composed of Tumor Stroma

In various solid tumors, including those of the breast, colon, lung, and prostate, tumor stroma including CAFs has been implicated in tumor growth, progression, angiogenesis, and metastasis [105,117]. The origin of CAFs has not been well defined, but Mishra et al. reported that bone marrow-derived MSCs could be a candidate cell origin for CAFs in solid tumors [118]. They showed that upon exposure to conditioned medium from human breast cancer MDAMB231 cells, MSCs became activated and exhibited a CAF-like myofibroblastic phenotype. Several reports demonstrated that circulating MSCs in the bloodstream are recruited and localized to developing tumors [119,120], suggesting that certain factors secreted from tumors recruit MSCs to solid tumors. Although the specific tumor-derived factors have not been identified, Wang et al. reported that the differentiation of MSCs into myofibroblasts is regulated by TGF-β [121]. Verona et al. reported that TGF-β stimulated prostatic stromal cells to express several genes related to myofibroblastic differentiation, including *COL11A1*, *TNC*, and *ACTA2*, and promoted reactive stroma formation and carcinoma growth in vivo [101]. These results suggest that MSCs, recruited by tumors and activated by TGF-β in solid tumors, may be a potential candidate source of CAFs.

Most human cancers result from an accumulation of somatic mutations arising in epithelial cells. The behavior of cancer cells is influenced by the tumor microenvironment including CAFs, ECM proteins, blood vasculature, and inflammatory cells. At present, it is not clear how cancer cells influence the generation of reactive stroma, or to what extent the reactive stroma is a result of phenotypic changes in resident cells versus cells recruited from other sites. 

**Figure 3:**
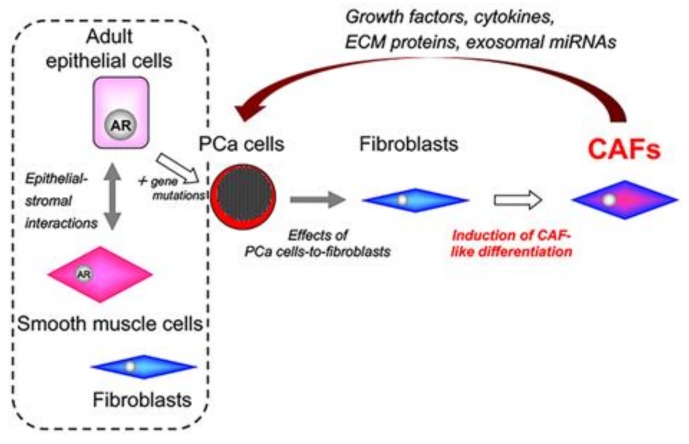
Induction of carcinoma-associated fibroblasts in the tumor stroma of prostate cancer. PCa cells induce CAF-like differentiation in normal fibroblasts. Importantly, heterogenous induction of CAF-like differentiation may be strongly dependent on the biochemical characteristics of PCa cells. AR: androgen receptor; CAFs: carcinoma-associated fibroblasts; PCa: prostate cancer.

The CAFs in tumor tissues are generated in response to specific paracrine signals from adjacent cancer cells. Although the origin of CAFs has not been determined, normal fibroblasts are abundant in prostate tissues. The variety of CAFs present may reflect different cell lineages or site-specific induction [108]. In PCa specimens, different malignant cell types are distributed heterogeneously throughout the tissues. This pathological feature led us to hypothesize that the generation of CAFs in PCa may be dependent on the biochemical characteristics of adjacent cancer cells. In our lab, Ishii et al. demonstrated that normal fibroblasts co-cultured with PCa cells become activated and exhibit biochemical characteristics of CAFs in a heterogenous manner [96]. Our results suggest that the heterogenous induction of CAF-like differentiation might be strongly dependent on the biochemical characteristics of adjacent cancer cells (Figure 3). In that study, we used three cancer cell lines for in vitro co-culture experiments: the androgen-sensitive LNCaP cell line and its derivative sublines, low-androgen-sensitive E9 cells, and androgen-insensitive AIDL cells. Co-cultures of commercially available prostate stromal cells and PCa cells in vitro changed the cytogenetic and biochemical profiles of stromal cells in a cancer cell line-specific manner. Our previous studies demonstrated that E9 and AIDL cells acquire a more aggressive phenotype in vitro and in vivo compared with the parental LNCaP cells [113,116,122,123]. However, we have not investigated in detail the differences in biochemical characteristics among these three cell lines. In our lab, we have shown that the biochemical characteristics of prostate stromal cells co-cultured with E9 cells, but not LNCaP or AIDL cells, resembled those of PCaSC-8 and PCaSC-9 cells [96]. This supports that heterogenous induction of a CAF-like phenotype may be strongly dependent on the specific characteristics of adjacent cancer cells. Identifying the mechanisms underlying the heterogenous induction of CAF-like differentiation in normal fibroblasts is an initial step toward designing CAF-targeted therapies for the treatment of PCa. Future studies will attempt to identify the specific profiles of the factors responsible for the CAF phenotype. 

## 10. Characteristics of Cell Populations Composed of Tumor Stroma

To investigate the effects of stromal components on tumorigenesis, Gleave et al. demonstrated that organ-specific fibroblasts were responsible for prostate tumor growth in vivo, i.e., LNCaP tumor formation was most induced by human bone fibroblasts, followed by rat UGM and Noble rat prostatic fibroblasts, but not by NIH-3T3, normal rat kidney, or human lung CCD16 fibroblasts [124]. In contrast, UGM has a normalizing effect on Dunning PCa cells [125]. Of interest, cancer cells in tumors have the ability to induce adjacent normal smooth muscle cells to exhibit a reactive myofibroblastic phenotype with some features similar to those of UGM [84]. Thus, although the tumor–stromal interactions are strongly complicated, it is obvious that the tumor and stroma affect each other and cooperate in tumor progression. 

Reactive stroma or CAFs adjacent to cancer cells secrete growth factors such as EGF family members, FGFs, insulin-like growth factors (IGFs), and TGF-β, which are involved in development and cancer progression, invasion, metastasis, and angiogenesis [102,126,127,128,129]. These growth factors are also produced by UGM [116,123,130]. UGM is composed of undifferentiated fibroblasts that induce instructive and permissive development and differentiation in the prostate. In our study, UGM shows androgen-dependent cell growth [116]. Interestingly, the expression of *FGF2* and *IGF1* mRNA was dramatically decreased in UGM cultured under androgen starvation, while *FGF7* mRNA was not influenced by androgen status. This suggests that FGF7/keratinocyte growth factor (KGF) may act as an androgen-independent stromal paracrine signal. Planz et al. reported that FGF7/KGF stimulated the proliferation of LNCaP cells in the presence of the anti-androgenic agent flutamide, showing that FGF7/KGF-induced cell proliferation in LNCaP cells is independent of cellular AR signaling [131]. Our results support the idea that androgen-independent stromal paracrine signaling by FGF7/KGF may bypass the functionally inactive AR and promote the proliferation of androgen-insensitive PCa cells during ADT.

Interestingly, Halin et al. demonstrated that androgen-insensitive PCa cells respond to castration when growing in an androgen-dependent prostate environment [132]. When androgen-insensitive AT-1 PCa cells were injected into the ventral prostates of Copenhagen rats, an androgen-dependent environment, castration reduced AT-1 tumor growth and vascular density in the tissue surrounding the tumor. These data demonstrated the importance of the cancer cell microenvironment for the action of androgens, i.e., the tumor growth of androgen-insensitive PCa cells was regulated by androgen-mediated stromal paracrine signals including growth factors, cytokines, and miRNAs. However, the mechanisms by which androgen-insensitive PCa cells respond to stromal paracrine signals under low-androgen conditions are not yet fully understood. 

The appearance of CAFs and ECM deposition in tumor tissues has been reported in many types of human cancers [108,109]. In particular, CAFs provide potentially oncogenic signals; CAF-derived tenascin-C and TGF-β participate in the acceleration of cancer cell invasion, and CAF-derived growth factors and angiogenic factor VEGF can stimulate cancer progression, including angiogenesis.

During primary cancer progression, CAFs communicate with cancer cells through the secretion of growth factors, cytokines, ECM proteins, and miRNAs [133,134]. For example, CAF-derived TGF-β, EGF, FGFs, VEGF, matrix metalloproteinases, and a number of other factors have been implicated in epithelial cancer progression [108,109,133,135]. Recently, Ishii et al. reported that CAFs derived from human lung cancer specimens retain their enhanced migratory activity for some time after separation from the cancer cells [136]. Thus, CAFs can maintain their ability to stimulate cancer progression, suggesting that inhibition of CAF generation in tumor tissues could be a new target for controlling primary cancer progression. 

As one candidate mediator from stromal components, secreted frizzled-related protein 1 has been found to promote tumor–stromal interactions in PCa [137]. More recently, Ao et al. reported that prostatic CAFs express high levels of stromal cell-derived factor-1 (also known as chemokine (C-X-C motif) ligand 12), and the stromal cell-derived factor-1/CXCR4 pathway between cancer cells and stromal cells could be involved in tumorigenesis of PCa [135].

ADT for patients with advanced PCa is intended to downregulate the concentration of circulating androgens or to block the transcriptional activation of AR [138]. Tumor stroma surrounding cancer cells is enriched in fibroblasts secreting AR-stimulating factors, VEGF, and TGF-β [139]. Previous studies have indicated that a number of growth factors and cytokines, including EGF, FGF7/KGF, IGF1, and IL-6, stimulate AR signaling and PSA expression in the context of androgen deficiency [139,140,141,142]. We have already reported that stromal remodeling after castration is accompanied by changes in the expression levels of these growth factors in the prostate [19]. Importantly, most fibroblastic cells in the prostate stroma are negative for AR [143,144], and the phenotypes of human PCa fibroblastic stromal cells are strongly heterogeneous [96]. Several studies have reported that androgen-sensitive and -insensitive interactions between cancer cells and stromal cells determine how PCa cells respond to androgen ablation [116,132]. In a low-androgen environment, aberrant activation between cancer cells and stromal cells may be an important mechanism controlling AR activity and AR-regulated PSA expression.

Soluble factors such as growth factors and cytokines derived from fibroblasts directly affect AR stimulation and AR-regulated PSA expression in the context of androgen deprivation. Shigemura et al. showed that conditioned medium from co-cultures of LNCaP cells and human prostate stromal fibroblasts induced PSA promoter reporter activity, ERK phosphorylation, and AR phosphorylation in LNCaP cells in vitro [145]. In our lab, Sasaki et al. also showed that fibroblasts directly affected PSA expression and the activation of Stat3, but not Akt or ERK, in LNCaP cells co-cultured in vitro [146]. In this study, we confirmed that EGF, IGF1, and IL-6 stimulated the expression of AR and PSA in LNCaP cells, suggesting that soluble factors derived from fibroblasts may function similarly to androgen in the absence of androgen. Thus, a heterogeneous combination of growth factors and cytokines derived from fibroblasts could be responsible for AR stimulation of PCa cells in the context of androgen deprivation.

## 11. Can We Discover New Biomarkers from Heterogeneous Stroma in PCa?

As described here, the tumor stroma is strongly heterogeneous. In our lab, Sasaki et al. formed a hypothesis regarding the role of fibroblasts in patients with PCa [146]. First, ‘protective’ fibroblasts prolong the duration of the blood supply in the tumors. Second, ‘protective’ fibroblasts cause the persistent stimulation of AR in PCa cells, preventing acute loss of AR function in PCa cells under ADT. In contrast, tumor-promoting ‘aggressive’ fibroblasts (i.e., CAFs) have also been identified [38]. CAFs surround cancer cells to support their survival and proliferation in a paracrine fashion. On the other hand, Hayashi et al. reported that rat UGM, which has features similar to CAFs but with the biological function of promoting the development, differentiation, and ultimately growth/quiescence of the prostate, elicited a reduction in the tumorigenic potential of Dunning prostatic adenocarcinoma [147]. Recent studies have also demonstrated that normal human fibroblasts can inhibit the proliferation of tumor cells [148,149]. We hypothesize that these ‘protective’ fibroblasts can also preserve the AR dependence of PCa cells under ADT. Moreover, Banerjee et al. demonstrated that epigenetic changes in prostatic fibroblasts caused DNA damage, mediating prostate tumor progression [150]. Thus, androgen deficiency may contribute to the reciprocal transfer of fibroblasts between the ‘protective’ and ‘aggressive’ states. Although Ayala et al. evaluated the potential of quantifying reactive stromal elements to predict disease progression [90], we suggest that the quality of fibroblasts is also an important factor that can be used to distinguish between ‘aggressive’ and ‘protective’ fibroblasts, which may be determined by evaluating a combination of stromal biomarkers. Future studies are needed to identify the specific profile of the stroma-derived factors responsible for disease progression. 

## 12. Role of CAF-Derived Exosomal miRNAs in Aberrant Activation of Tumor–Stromal Interactions

Tumor–stromal interactions are a dynamic process that involves not only direct cell–cell contact via cell surface adhesion molecules, but also indirect cell–cell communication via soluble factors. Activated fibroblasts such as CAFs communicate with adjacent cancer cells via soluble factors secreted into the extracellular space [151,152]. Of course, this is only one mechanism of tumor–stromal interactions, and others may exist in parallel, such as membrane-derived exosomes. In addition to soluble factors, membrane-derived exosomes have been reported to modulate tumor progression [134].

In cancer, CAFs aberrantly secrete large amounts of exosomes to transport stromal paracrine signals including miRNAs within the tumor microenvironment [134]. Exosomes are membrane-enclosed extracellular vesicles (EVs) that contribute to tumor progression. EVs are classified into three main types according to size and biogenesis: exosomes (30–100 nm), microvesicles (100–1000 nm), and oncosomes (1–10 µm). The discovery of exosomes provides novel insights into indirect cell–cell communication between cancer cells and CAFs. Exosomes play critical roles in several aspects of tumor progression, including growth, invasion, angiogenesis, and metastasis. Exosomes contain a wide variety of bioactive molecules including signal peptides, lipids, miRNAs, mRNAs, and DNA [134]. Interestingly, miRNAs are stably transferred by exosomes to recipient cells where they modulate gene expression, resulting in functional effects on cell fate. 

miRNAs are small (~20–23 nucleotides) non-coding RNAs that negatively regulate the expression of specific target genes, including tumor suppressors and oncogenes, at the transcriptional and translational levels [153]. In addition, target genes of miRNAs affect the fates of recipient cells including proliferation, differentiation, adhesion, and migration, e.g., miRNAs reciprocally regulate TGF-β signaling during tumor progression [154]. CAF-derived exosomal miRNAs affect the cell fates of recipient cancer cells. In addition to soluble factors such as growth factors, cytokines, and ECM proteins, exosomal miRNAs play a critical role in indirect cell–cell communication. Recently, several studies have reported that the inhibition of miRNAs can serve as a novel therapeutic strategy for treating or effectively managing PCa [155,156,157,158]. 

In addition to the role of miRNAs as therapeutic targets, circulating exosomal miRNAs have potential as liquid biopsy and noninvasive biomarkers for early detection and diagnosis [134]. Exosomes are also found abundantly in bodily fluids including blood, urine, cerebrospinal fluid, breast milk, saliva, ascites fluid, and amniotic fluid [159,160]. The composition of miRNAs in circulating exosomes is similar to that found in their originating cells [161,162]. In contrast, Schageman et al. reported that levels of certain RNA sequences were substantially different between exosomes and the parental serum samples [163]. These reports suggest the possibility of using disease-specific exosomal miRNA signatures in bodily fluids as unique diagnostic markers.

Regarding the use of miRNA expression signatures as biomarkers, several studies have recently reported specific miRNA expression profiles in different bodily fluid samples derived from patients with BPH or PCa (Table 1). Importantly, Cochetti et al. reported that different levels of serum miRNAs between PCa and BPH may be reliable candidates for developing minimally invasive biomarkers as diagnostic and prognostic tools [164]. In general, serum PSA is currently the most useful biomarker to detect PCa, whereas an increase in serum PSA levels is also observed in BPH or inflammation in the prostate. PSA is an androgen-regulated serine protease produced in both normal luminal epithelial cells and well-differentiated PCa cells. ADT for patients with advanced PCa is intended to downregulate the concentration of circulating androgens or to block AR signals, leading to a reduction in serum PSA levels. In our previous work, Sasaki et al. found that the PSA kinetics after ADT were not an accurate prognostic marker when we regarded serum PSA levels after ADT as the number of viable cancer cells [165,166].

In contrast to normal luminal epithelial cells or well-differentiated PCa cells, most fibroblasts are AR negative. In addition, the stromal compartment of a tumor is genetically more stable than the cancer compartment [109]. Several studies have demonstrated changes in miRNA expression in the stromal compartment of PCa [92,167,168]. For example, Josson et al. demonstrated that miR-409-3p was significantly elevated in the stroma of patients with a high (>7) versus low Gleason score (<7) [168]. Ren et al. identified 299 miRNAs that were differentially expressed between high-grade/stage and low-grade/stage PCa groups, suggesting that PCa can be readily classified as high grade/stage and low-grade/stage according to its global miRNA expression signature [169]. In the future, the identification of CAF-derived exosomal miRNA signatures may provide novel prognostic information reflecting the grade/stage of PCa under ADT treatment.

## 13. Concluding Remarks

An interesting feature of the adult prostate is that prostate tissue does not actively proliferate, and there is little cellular turnover, even in the presence of growth-stimulating androgens. The reason for this is that stromal paracrine signals (morphogens) derived from smooth muscle cells function to maintain the functional differentiation and growth/quiescence of epithelial cells. In proliferative diseases of the aging human prostate such as BPH and PCa, however, alterations of stromal structure and components occur, i.e., increased numbers of fibroblasts and myofibroblasts but a decreased number of smooth muscle cells, referred to as stromal remodeling. Stromal remodeling-induced deregulation of epithelial–stromal interactions is considered to be responsible for the initiation and/or promotion of proliferative prostatic diseases because of aberrant growth-stimulatory signals (mitogens). Therefore, maintaining stromal structure and components in the aging human prostate is a critical factor to reduce the incidence of proliferative prostatic diseases.

## Figures and Tables

**Table 1:** Latest studies of miRNA expression profiles in different body samples derived from patients with BPH or PCa.

Source	Prostatic Disease	Reference
Tissue	BPH	[170]
Tissue	BPH and PCa	[171]
Tissue	BPH and PCa	[172]
Tissue	BPH and PCa	[173]
Plasma EVs	BPH and PCa	[174]
Serum exosomes	BPH and PCa	[175]
Urine	BPH and PCa	[176]
Urinary EVs	BPH and PCa	[177]

EVs: extracellular vesicles; BPH: benign prostatic hyperplasia; PCa: prostate cancer.
